# Prognostic relevance of gait-related cognitive functions for dementia conversion in amnestic mild cognitive impairment

**DOI:** 10.1186/s12877-023-04175-8

**Published:** 2023-07-31

**Authors:** Cosimo Tuena, Sara Maestri, Silvia Serino, Elisa Pedroli, Marco Stramba-Badiale, Giuseppe Riva, the Alzheimer’s Disease Neuroimaging Initiative, Lisa C. Silbert, Betty Lind, Rachel Crissey, Jeffrey A. Kaye, Raina Carter, Sara Dolen, Joseph Quinn, Lon S. Schneider, Sonia Pawluczyk, Mauricio Becerra, Liberty Teodoro, Karen Dagerman, Bryan M. Spann, James Brewer, Adam Fleisher, Helen Vanderswag, Jaimie Ziolkowski, Judith L. Heidebrink, Lisa Zbizek-Nulph, Joanne L. Lord, Colleen S. Albers, Ronald Petersen, Sara S. Mason, David Knopman, Kris Johnson, Javier Villanueva-Meyer, Valory Pavlik, Nathaniel Pacini, Ashley Lamb, Joseph S. Kass, Rachelle S. Doody, Victoria Shibley, Munir Chowdhury, Susan Rountree, Mimi Dang, Yaakov Stern, Lawrence S. Honig, Akiva Mintz, Beau Ances, John C. Morris, David Winkfield, Maria Carroll, Georgia Stobbs-Cucchi, Angela Oliver, Mary L. Creech, Mark A. Mintun, Stacy Schneider, David Geldmacher, Marissa Natelson Love, Randall Griffith, David Clark, John Brockington, Daniel Marson, Hillel Grossman, Martin A. Goldstein, Jonathan Greenberg, Effie Mitsis, Raj C. Shah, Melissa Lamar, Patricia Samuels, Ranjan Duara, Maria T. Greig-Custo, Rosemarie Rodriguez, Marilyn Albert, Chiadi Onyike, Leonie Farrington, Scott Rudow, Rottislav Brichko, Stephanie Kielb, Amanda Smith, Balebail Ashok Raj, Kristin Fargher, Martin Sadowski, Thomas Wisniewski, Melanie Shulman, Arline Faustin, Julia Rao, Karen M. Castro, Anaztasia Ulysse, Shannon Chen, P. Murali Doraiswamy, Jeffrey R. Petrella, Olga James, Terence Z. Wong, Salvador Borges-Neto, Jason H. Karlawish, David A. Wolk, Sanjeev Vaishnavi, Christopher M. Clark, Steven E. Arnold, Charles D. Smith, Gregory A. Jicha, Riham El Khouli, Flavius D. Raslau, Oscar L. Lopez, MaryAnn Oakley, Donna M. Simpson, Anton P. Porsteinsson, Kim Martin, Nancy Kowalski, Melanie Keltz, Bonnie S. Goldstein, Kelly M. Makino, M. Saleem Ismail, Connie Brand, Gaby Thai, Aimee Pierce, Beatriz Yanez, Elizabeth Sosa, Megan Witbracht, Brendan Kelley, Trung Nguyen, Kyle Womack, Dana Mathews, Mary Quiceno, Allan I. Levey, James J. Lah, Ihab Hajjar, Jeffrey M. Burns, Russell H. Swerdlow, William M. Brooks, Daniel H. S. Silverman, Sarah Kremen, Liana Apostolova, Kathleen Tingus, Po H. Lu, George Bartzokis, Ellen Woo, Edmond Teng, Neill R. Graff-Radford, Francine Parfitt, Kim Poki-Walker, Martin R. Farlow, Ann Marie Hake, Brandy R. Matthews, Jared R. Brosch, Scott Herring, Christopher H. van Dyck, Adam P. Mecca, Susan P. Good, Martha G. MacAvoy, Richard E. Carson, Pradeep Varma, Howard Chertkow, Susan Vaitekunas, Chris Hosein, Sandra Black, Bojana Stefanovic, Chris Heyn, Ging-Yuek Robin Hsiung, Ellen Kim, Benita Mudge, Vesna Sossi, Howard Feldman, Michele Assaly, Elizabeth Finger, Stephen Pasternak, Irina Rachinsky, Andrew Kertesz, Dick Drost, John Rogers, Ian Grant, Brittanie Muse, Emily Rogalski, Jordan Robson, M.-Marsel Mesulam, Diana Kerwin, Chuang-Kuo Wu, Nancy Johnson, Kristine Lipowski, Sandra Weintraub, Borna Bonakdarpour, Nunzio Pomara, Raymundo Hernando, Antero Sarrael, Howard J. Rosen, Bruce L. Miller, Micheal W. Weiner, David Perry, Raymond Scott Turner, Kathleen Johnson, Brigid Reynolds, Kelly MCCann, Jessica Poe, Gad A. Marshall, Reisa A. Sperling, Keith A. Johnson, Jerome Yesavage, Joy L. Taylor, Steven Chao, Jaila Coleman, Jessica D. White, Barton Lane, Allyson Rosen, Jared Tinklenberg, Christine M. Belden, Alireza Atri, Bryan M. Spann, Kelly A. Clark, Edward Zamrini, Marwan Sabbagh, Ronald Killiany, Robert Stern, Jesse Mez, Neil Kowall, Andrew E. Budson, Thomas O. Obisesan, Oyonumo E. Ntekim, Saba Wolday, Javed I. Khan, Evaristus Nwulia, Sheeba Nadarajah, Alan Lerner, Paula Ogrocki, Curtis Tatsuoka, Parianne Fatica, Evan Fletcher, Pauline Maillard, John Olichney, Charles DeCarli, Owen Carmichael, Vernice Bates, Horacio Capote, Michelle Rainka, Michael Borrie, T.-Y. Lee, Rob Bartha, Sterling Johnson, Sanjay Asthana, Cynthia M. Carlsson, Allison Perrin, Anna Burke, Douglas W. Scharre, Maria Kataki, Rawan Tarawneh, Brendan Kelley, David Hart, Earl A. Zimmerman, Dzintra Celmins, Delwyn D. Miller, Laura L. Boles Ponto, Karen Ekstam Smith, Hristina Koleva, Hyungsub Shim, Ki Won Nam, Susan K. Schultz, Jeff D. Williamson, Suzanne Craft, Jo Cleveland, Mia Yang, Kaycee M. Sink, Brian R. Ott, Jonathan Drake, Geoffrey Tremont, Lori A. Daiello, Jonathan D. Drake, Marwan Sabbagh, Aaron Ritter, Charles Bernick, Donna Munic, Akiva Mintz, Abigail O’Connelll, Jacobo Mintzer, Arthur Wiliams, Joseph Masdeu, Jiong Shi, Angelica Garcia, Marwan Sabbagh, Paul Newhouse, Steven Potkin, Stephen Salloway, Paul Malloy, Stephen Correia, Smita Kittur, Godfrey D. Pearlson, Karen Blank, Karen Anderson, Laura A. Flashman, Marc Seltzer, Mary L. Hynes, Robert B. Santulli, Norman Relkin, Gloria Chiang, Athena Lee, Michael Lin, Lisa Ravdin

**Affiliations:** 1 Applied Technology for Neuro-Psychology Lab, IRCCS Istituto Auxologico Italiano, Milan, Italy; 2 Department of Psychology, Università degli Studi Milano-Bicocca, Milan, Italy; 3 Faculty of Psychology, Università eCampus, Novedrate, Italy; 4 Department of Geriatrics and Cardiovascular Medicine, IRCCS Istituto Auxologico Italiano, Milan, Italy; 5 Humane Technology Lab, Università Cattolica del Sacro Cuore, Milan, Italy

**Keywords:** Trail making test, Digit symbol substitution test, Embodiment, Cognitive dysfunction, Gait abnormalities, Gait assessment

## Abstract

**Background:**

Increasing research suggests that gait abnormalities can be a risk factor for Alzheimer’s Disease (AD). Notably, there is growing evidence highlighting this risk factor in individuals with amnestic Mild Cognitive Impairment (aMCI), however further studies are needed. The aim of this study is to analyze cognitive tests results and brain-related measures over time in aMCI and examine how the presence of gait abnormalities (neurological or orthopedic) or normal gait affects these trends. Additionally, we sought to assess the significance of gait and gait-related measures as prognostic indicators for the progression from aMCI to AD dementia, comparing those who converted to AD with those who remained with a stable aMCI diagnosis during the follow-up.

**Methods:**

Four hundred two individuals with aMCI from the Alzheimer’s Disease Neuroimaging Initiative (ADNI) database were included. Robust linear mixed-effects models were used to study the impact of gait abnormalities on a comprehensive neuropsychological battery over 36 months while controlling for relevant medical variables at baseline. The impact of gait on brain measures was also investigated. Lastly, the Cox proportional-hazards model was used to explore the prognostic relevance of abnormal gait and neuropsychological associated tests.

**Results:**

While controlling for relevant covariates, we found that gait abnormalities led to a greater decline over time in attention (DSST) and global cognition (MMSE). Intriguingly, psychomotor speed (TMT-A) and divided attention (TMT-B) declined uniquely in the abnormal gait group. Conversely, specific AD global cognition tests (ADAS-13) and auditory-verbal memory (RAVLT immediate recall) declined over time independently of gait profile. All the other cognitive tests were not significantly affected by time or by gait profile. In addition, we found that ventricles size increased faster in the abnormal gait group compared to the normal gait group. In terms of prognosis, abnormal gait (HR = 1.7), MMSE (HR = 1.09), and DSST (HR = 1.03) covariates showed a higher impact on AD dementia conversion.

**Conclusions:**

The importance of the link between gait and related cognitive functions in terms of diagnosis, prognosis, and rehabilitation in aMCI is critical. We showed that in aMCI gait abnormalities lead to executive functions/attention deterioration and conversion to AD dementia.

**Supplementary Information:**

The online version contains supplementary material available at 10.1186/s12877-023-04175-8.

## Introduction

Walking is a physiological milestone of normal human neurodevelopment as well as a crucial part of daily life, and it is no longer regarded solely as a physical factor [1, 2]. Indeed, gait control is a complex process that involves the integration of motor, perceptual, and cognitive processes [2]. Specifically, the executive functions, including attentional control, cognitive flexibility, psychomotor processing, inhibition, and goal setting, share with motor abilities and gait control a widespread brain network of prefrontal cortical and subcortical regions [3]. These include the prefrontal cortex, the medial temporal lobe, and the nigrostriatal system but also the size of ventricles, cerebellum, white matter tracts, and the parietal lobes [2, 4, 5].

A consistent body of studies reported a relationship between gait abnormalities and early signs of cognitive decline among cognitively healthy participants [2, 6, 7]. Gait abnormalities include disorders that result in slow, unsteady, staggering, shuffling, and/or asymmetrical walking due to neurological, musculoskeletal, and/or other acquired medical conditions [8–10]. Disorders of gait can be evaluated through clinical visual inspection or through quantitative parameters (e.g., speed, stride length, swing, and stance time) that reflect the observed gait abnormality [11]. The former is a useful and reliable method in everyday clinical practice, whereas the latter requires technological equipment that can be used to further differentiate individuals according to their cognitive status [8, 11].

For instance, there is considerable evidence showing that gait abnormalities could predict a cognitive decline over time measured with the Digit Symbol Substitution Test (DSST) [6, 7, 12–16], a measure of psychomotor speed and attention [17]. Furthermore, gait abnormalities predict the decline in divided attention and cognitive flexibility, as measured with the Trail Making Test part B (TMT-B) [6, 7]. The presence of gait abnormalities is also longitudinally associated with a decline in global cognition tests [6, 7]. Indeed, gait disorders have been identified as one of the factors associated with the development of dementia [8].

Early evidence by Camicioli and colleagues [18] found that slow gait is evident on clinical examination before or coincident with the development of cognitive impairment in healthy older people. Even though some diagnostic criteria include the presence of gait disturbances in the exclusion criteria of Alzheimer’s disease (AD) [19], a recent meta-analysis [20] suggested that gait performance predicts AD dementia (Hazard Ratio—HR = 1.03). The longitudinal study of Kuate-Tegueu and co-authors [21] showed that gait speed (HR = 1.2) and Trail Making Test part A (HR = 1.4; TMT-A), which requires complex visual scanning and psychomotor speed [22], were associated with incident AD. This is consistent with a recent study showing that gait abnormalities (slower gait speed, lower cadence, longer double support time, and greater stance time variability) have been associated with AD neuropathology (i.e., beta-amyloid) in cognitively healthy older individuals [23]. Another study [24] showed that in older people, cerebral deposition of beta-amyloid is associated with slower gait speed and lower limbs functioning. A recent large multi-database study [25] showed that higher gait variability can discriminate AD from other neurodegenerative diseases (e.g., Parkinson’s disease, frontotemporal dementia, dementia with Lewy bodies). The authors concluded that high gait variability could be a marker for cortical-related cognitive dysfunctions which alter both cognition and gait control.

The concept of Mild Cognitive Impairment (MCI) has offered a unique window to study the development of AD. MCI is the transitional condition between normal and pathological cognitive aging [26]. In particular, the amnestic MCI (aMCI) type, namely individuals who experience more memory loss than expected for their age and education and are more likely to develop AD than the non-amnestic type (naMCI), has received increasing attention in the last decade [26]. In patients with MCI, the prevalence of slow gait or neurological gait abnormalities reaches 46%, almost threefold higher than in healthy older adults without MCI; in addition, neurological gait disorders were more common in patients with aMCI than in those with naMCI [11]. Interestingly, a growing body of studies revealed that gait disorders may be a risk factor for cognitive deterioration in this population. For instance, Doi and colleagues [27] found that patients with MCI and slow gait reported greater cognitive deficits on a comprehensive neuropsychological battery, including the Mini-Mental State Examination (MMSE), DSST, TMT-A, and TMT-B, compared to MCI without slow gait, healthy older people with slow gait and without slow gait.

Literature showed different longitudinal studies on MCI or aMCI population, in which the influence of gait abnormalities on cognition was analysed. Buracchio and colleagues [28] demonstrated that a decline in gait speed occurred about 12 years before MCI, therefore it may be a sensitive marker of cognitive change. Furthermore, individuals with slow gait had 7 times the risk of progressing to dementia and a higher attributable risk than those with cognitive decline alone, who had 3 times the risk of progressing [29]. Another study showed that slower maximum walking speed and longer time on the Test Timed Up and Go test were predictive of cognitive decline, as assessed according to the Montreal Cognitive Assessment-Japan score decline [30]. Evidence indicated that aMCI who developed AD had lower gait speed than those who did not develop AD. Both gait speed and gait variability could be markers to early identify aMCI at risk to progress to AD [31]. Also, the study of Tian and co-authors [32] confirmed that slower baseline gait speed was associated with a higher hazard of developing aMCI/AD. A study [33] showed that the presence of at least one copy of apolipoprotein E polymorphism ε4 allele in MCI is longitudinally associated with a decline in both gait performance and global cognition. Intriguingly, one randomized controlled trial [34] showed that administering donepezil to improve cholinergic neurotransmission in MCI improves gait speed during dual-task, possibly due to an enhancement in frontal functions.

Despite convincing evidence that specific gait parameters can be a risk factor for dementia conversion, no previous studies have investigated which neuropsychological tests would show a greater decline among aMCI patients with and without gait disorders, and what is the prognostic relevance of gait and related neuropsychological functions.

We want to explore if individuals with abnormal gait at the beginning of the study due to neurological (e.g., slow, broad-based, unsteady, stooped, or asymmetrical gait) or musculoskeletal (e.g., injury, pain) deficits, will show a steeper decline on a set of neuropsychological tests, possibly the ones that assess, in addition to global cognition, psychomotor speed, attention, and/or executive functions. In addition, we expect that these findings are the result of the gait profile (i.e., abnormal vs. normal) itself and its possible neural altered mechanisms (e.g., AD pathology) rather than functional, medical (e.g., cerebrovascular accidents, multimorbidity, polypharmacy), and cognitive confounding factors at baseline. Secondarily, we also wanted to explore if the presence of gait disorders is associated with gait related brain measures (e.g., medial temporal regions volume, ventricles size, brain metabolism) Lastly, we want to explore what is the prognostic impact of gait disorders and the significantly affected tests on conversion to AD dementia in aMCI.

## Methods

### Study sample

Data used in the preparation of this article were obtained from the Alzheimer’s Disease Neuroimaging Initiative (ADNI) database (adni.loni.usc.edu). The ADNI was launched in 2003 as a public–private partnership, led by Principal Investigator Dr. Michael W. Weiner, MD. The primary goal of ADNI has been to test whether serial magnetic resonance imaging (MRI), positron emission tomography (PET), other biological markers, and clinical and neuropsychological assessment can be combined to measure the progression of MCI and early AD. In particular, we used the ADNI phase 1 database with a total of 402 participants (recruited in the North America; https://www.adni3.org/locations). 210 (52%) aMCI individuals did not convert to dementia from baseline to the last time-point considered (36 months) in this study; 174 (43%) converted to dementia and 18 (5%) reverted to normal cognition during the follow-up window. Other socio-demographic and clinical characteristics are shown in Table 1.

**Table 1:** Summary of socio-demographic and medical variables at baseline of the aMCI group by gait profiles

	Abnormal gait = 39			Normal gait = 363			
	N	Mean	SD	N	Mean	SD	*p*-value
Age (years)	39	77.93	6.47	363	74.42	7.38	**0.003**
Gender	39			363			0.123
Female	9	23%		134	37%		
Male	30	77%		229	63%		
Education (years)	39	16.03	3.12	363	15.6	3.02	0.388
Ethnic group	39			363			0.751
Am. Indian/Alaskan	0	0%		1	0%		
Asian	1	3%		8	2%		
Black	0	0%		15	4%		
More than one	0	0%		1	0%		
White	38	97%		338	93%		
Marital status	39			363			0.114
Divorced	0	0%		25	7%		
Married	31	79%		291	80%		
Never married	2	5%		5	1%		
Widowed	6	15%		42	12%		
FAQ (points)	39	5.72	4.62	360	3.68	4.42	**0.001**
BMI (kg/m^2^)	39	27.06	3.81	362	25.95	3.9	0.069
Polypharmacy (n°)	39	8.38	4.1	363	7.55	4.47	0.191
Medical conditions (n°)	39	7.95	2.34	363	6.3	2.52	** < 0.001**
GDS (points)	39	1.72	1.41	363	1.56	1.37	0.498
ApoE4 alleles	39			363			0.184
0	23	59%		164	45%		
1	14	36%		154	42%		
2	2	5%		45	12%		
FDG-PET (SUV)	16	1.13	0.11	189	1.2	0.13	**0.027**
HP (mm^3^)	28	6,051.43	1,099.64	292	6,432.45	1,069.97	0.051
MTL (mm^3^)	28	18,020.93	2,717.03	292	18,742.46	3,016.69	0.150
V (mm^3^)	38	55,807.34	28,637.46	356	43,368.73	22,861.64	**0.011**
WMH (cm^3^)	38	1.18	2.59	363	0.87	2.76	0.084
CVA (n°)	38	0.21	0.58	359	0.11	0.63	0.054
Gait type	39			363			** < 0.001**
Neurologic	20	51%		0	0%		
Normal	0	0%		363	100%		
Orthopedic	19	49%		0	0%		

*aMCI* Amnestic Mild Cognitive Impairment, *BMI* Body Mass Index, *GDS* Geriatric Depression Scale, *FAQ* Functional Activities Questionnaires, *ApoE4* Apolipoprotein E4, *CVA* Cerebrovascular Accidents, *FDG-PET* average metabolism FDG-PET of angular, temporal, and posterior cingulate cortices, *HP* Hippocampal Volume, *MLT* Medial Temporal Lobe volume, *SUV* Standardized Uptake Value for regional cerebral metabolic rate of glucose, *V* Ventricles volume, *WMH* White Matter Hyperintensities volume. Mean and SD are reported. Bold values represent significant *p*-values

In the ADNI protocol [35], MCI individuals were diagnosed according to the Petersen criteria [26] of aMCI (in this study, both single and multiple domain aMCI are considered). In the aMCI group, participants were included if: there was a memory complaint by subject or caregiver that is verified by a study partner; abnormal memory function documented by scoring below the education adjusted cutoff on the logical memory II subscale from the Wechsler memory scale – revised; MMSE score was between 24 and 30; Clinical Dementia Rating (CDR) was 0.5; general cognition and functional performance were sufficiently preserved such that a diagnosis of AD cannot be made by the site physician at the time of the screening visit; the modified Hachinski score was ≤ 4; they had an age between 55 and 90 years old; they had permitted medications stable for at least 4 weeks prior to screening; if the Geriatric Depression Scale (GDS) [36] score was < 6; they had adequate visual and auditory acuity to allow neuropsychological testing and good general health with no additional diseases; willing and able to complete all baseline assessments and to participate in a 3-year protocol; willing to undergo MRI 1.5 Tesla neuroimaging and provide DNA for ApoE assessments and banking as well as plasma samples at protocol specified time points; completed 6 grades of education (or had a good work history sufficient to exclude mental retardation); fluent in English or Spanish.

MCI participants were excluded if there was any significant neurologic disease other than suspected incipient AD or history of significant head trauma followed by persistent neurologic defaults or known structural brain abnormalities; evidence of infection, infarction, or other focal lesions, multiple lacunes or lacunes in a critical memory region; presence of pacemakers, aneurysm clips, artificial heart valves, ear implants, metal fragments or foreign objects in the eyes, skin or body; major depression, bipolar disorder within the past 1 year, psychotic features, agitation or behavioral problems within the last 3 months, history of schizophrenia; history of alcohol or substance abuse or dependence within the past 2 years; any significant systemic illness or unstable medical condition; clinically significant abnormalities in vitamin B12, rapid plasma regain test, or thyroid function tests; residence in skilled nursing facility; current use of specific psychoactive medications and warfarin; participation in clinical studies involving neuropsychological measures being collected more than one time per year. This information was extracted from the ADNI phase 1 [35] clinical protocol section (https://adni.loni.usc.edu/methods/documents/).

Ethical approval for data collection and sharing was given by the institutional review boards of the participating institutions in the ADNI.

### Gait screening and medical baseline measurements

Neurological gait examination was carried out according to the ADNI clinical protocol by licensed specialists at screening visits to ensure patient eligibility before the baseline assessment. The ADNI specialist determined whether gait was normal or abnormal after visual inspection of gait patterns (e.g., walking for a short distance) and balance (i.e., tandem walk, Romberg test). Gait examination for each patient was described by the ADNI specialist and this information was retrieved from the neurological examination ADNI file (NEUROEXM.csv). Patients were categorized, according to the description reported in this file, independently by two authors (C.T., S.M.) as having orthopedic (e.g., antalgic, orthopedic injury/surgery, arthritis, musculoskeletal problems), neurologic (e.g., broad-based, slow, unsteady gait, positive Romberg, difficulty with the tandem walk, different arms swing), or unclear (e.g., mixed, unable to determine) gait using the sign-based approach table proposed by Nonnekes and co-authors [37]. To exclude any confounding effect of cerebrovascular lesion on gait examination, white matter hyperintensities and the number of cerebrovascular accidents at screening visit were included (see Table 1).

Baseline assessment included the number of medications taken (including integrators), the number of medical conditions in the patient’s history before the screening visit, functional activities questionnaires (FAQ; [38], GDS, body mass index (BMI), as well as ADNI phase 1 brain-related measured. These included hippocampal, medial temporal lobe, and ventricles volumes and average metabolism of angular, temporal, and posterior cingulate regions assessed by FDG-PET (fluorodeoxyglucose positron emission tomography) (see Table 1).

MRI data (hippocampi, medial temporal lobes, and ventricles) were provided and structures volumes were computed by ADNI specialists as reported in the MRI methods web-page (https://adni.loni.usc.edu/methods/mri-tool/mri-analysis/). Similarly, FDG-PET data were provided and computed by ADNI PET specialists (https://adni.loni.usc.edu/methods/pet-analysis-method/pet-analysis/). As described by ADNI PET Core, FDG-PET was computed with a meta-ROI (regions of interests) method of 5 AD-related cortices of the brain (right and left temporal, right and left angular, and posterior cingulate) [39].

### Longitudinal measurements of cognitive functions and brain imaging

Eight neuropsychological tests were considered for the analyses. Tests were administered at baseline (0), 6, 12, 18, 24, and 36 months. These included: global cognition (MMSE, [40]; Alzheimer’s Disease Assessment Scale-Cognitive 13 – ADAS-13, [41]), constructional apraxia (Clock Drawing Test – CDT, [42]), working memory (Digit Span Backward – DSB, [17]), short-term memory (Digit Span Forward – DSF, [17]), long-term memory (Rey Auditory Verbal Learning Test – RAVLT, [43]), psychomotor speed and attention (DSST, [17]), psychomotor speed and visual search (TMT-A, [22]), and divided attention (TMT-B, [22]). See Supplementary Material 1 for test description. Table 2 shows descriptive statistics of baseline neuropsychological tests in the two populations by gait profiles. Secondarily, we examined the longitudinal changes in the hippocampi, medial temporal lobe, ventricles size, and FDG-PET (right and left temporal, right and left angular, and posterior cingulate metabolism). See Table 1 for baseline characteristics of these variables in the two groups.

**Table 2:** Results of baseline neuropsychological tests in the two populations by gait profiles

	aMCI		
	Abnormal gait	Normal gait	*p*-value
MMSE (points)	26.62 (1.65)	27.06 (1.79)	0.138
ADAS-13 (points)	20.44 (6.59)	11.40 (4.37)	0.112
RAVLT Immediate (points)	28.05 (8.43)	30.90 (9.09)	0.087
RAVLT Learning (points)	3.15 (2.47)	3.29 (2.34)	0.658
RAVLT Forgetting (points)	4.67 (1.85)	4.66 (2.28)	0.793
DSST (points)	35.15 (8.86)	37.00 (11.37)	0.327
CDT (points)	3.95 (1.07)	4.19 (0.99)	0.141
DSF (points)	6.44 (1.02)	6.53 (1.09)	0.567
DSB (points)	4.26 (0.92)	4.59 (1.13)	0.077
TMT-A (seconds)	42.54 (13.24)	45.08 (23.48)	0.690
TMT-B (seconds)	139.44 (67.99)	130.55 (74.15)	0.273

*aMCI* Amnestic Mild Cognitive Impairment, *MMSE* Mini-Mental State Examination, *ADAS-13* Alzheimer’s Disease Assessment Scale-Cognitive 13, *RAVLT* Rey Auditory Verbal Learning Test, *DSST* Digit Symbol Substitution Test, *CDT* Clock Drawing Test, *DSF* Digit Span Forward, *DSB* Digit Span Backward, *TMT-A* Trail Making Test part A, *TMT-B* Trail Making Test part B. Mean and SD are reported. For numerical variables, an F-test is performed, while for categorical variables, a Chi-squared test is used

### Statistical analysis

Statistical analyses were done with R software (v. 3.6.3). To explore the trends of neuropsychological functions over time according to gait profiles we used regression as in a similar study [6]. To handle unbalanced groups, missing values, and violations of linear model assumptions, separate Robust Linear Mixed-Effects Models (RLMM) were used to study the impact of gait on each cognitive measure for the whole aMCI group (*N* = 402), regardless of the conversion status during the follow-up. A nested random effect formula with random intercept [cognitive test ~ gait profile*time + covariate^1^ + covariate^2^ + covariate^n^ + (1|Site ID:Patient ID)] was used to account for the hierarchical structure of patients assessed in different medical sites. *P*-value significance of RLMM was calculated as suggested by Geniole and co-authors [44]. R package used for RLMM was *robustlmm* [45]. Numerical variables of all the regressions were standardized (z-score) to improve estimates interpretation. The Intra-class Correlation Coefficient (ICC) was used as a measure for the variance explained by the random effects.

Lastly, Cox regression model, using the *survival* package [46], was used to see which of the significant cognitive tests (see Results sections) and gait profiles (normal vs. abnormal) had a significant prognostic relevance for dementia conversion in aMCI. For this analysis, we included only stable aMCI (non-converter) which had all the longitudinal visits until month-36 (*N* = 128) and converter aMCI which developed AD dementia during the follow-up (*N* = 174). aMCI which reverted to normal cognition were not included in this analysis (*N* = 18). Stable aMCI were patients who had a stable diagnosis during the 36 months according to Petersen criteria [26]; reverted aMCI were participants who no longer satisfied such criteria and reverted to a normal cognitive status during the follow-up period. As a higher DSST score represent better performance and, in the TMT-AB a higher score indicates worse functioning, we reversed the DSST score with the formula (the maximum possible score of 93-patient’s score) and the MMSE score with the formula (the maximum possible score of 30-patient’s score) to make the HR comparable to the HR direction of the TMT-AB.

To evaluate differences between the two gait profiles, non-parametric tests were used due to unbalanced groups (Wilcoxon rank-sum test for numeric dependent variables and Chi-square test for categorical variables). Variables with significant differences between the gait groups were used as covariates in the aMCI group (see sample differences in Tables 1 and 2). Age, FAQ, number of medical conditions, volumes of the ventricles, and FDG-PET metabolism of angular, temporal, and posterior cingulate regions were put as covariates in each model. In addition, for brain-related measures (hippocampal, medial temporal lobe, and ventricles volumetry and FDG-PET) we adjusted the four RLMM for the baseline values (e.g., hippocampal RLMM adjusted for age, FAQ, number of medical conditions, volumes of the ventricles, FDG-PET, and baseline hippocampi volumetry) in the two gait profiles. The significance level for all the analyses was set to 0.05.

## Results

Tables 1 and 2 provide a summary of baseline socio-demographics and clinical measures. The abnormal gait group were older, had greater functional impairment, more medical conditions, reduced FDG-PET metabolism, and larger ventricles. Conversely no difference was observed in the cognitive tests reported in Table 2.

### Effect of gait profiles on cognition

**Fig. 1:**
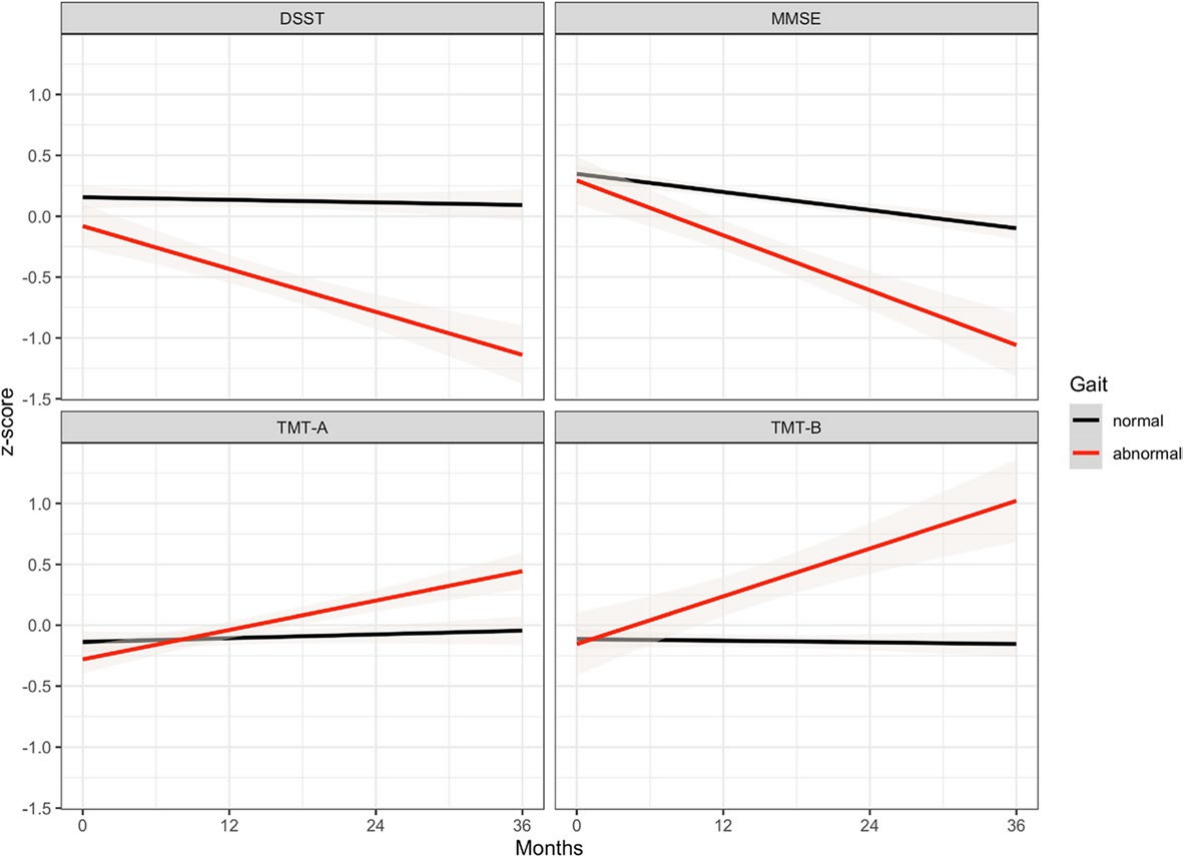
Trends in the amnestic Mild Cognitive Impairment (aMCI) group by gait profile. A negative z-score for The Digit Symbol Substitution Test (DSST) and Mini-Mental State Examination (MMSE) represents lower scores, conversely, a positive z-score for the Trail-Making Test Part A and B (TMT-A, TMT-B) represents higher completion times. Gray shades in the plot represent the standard error of the regression line

RLMM regressions with covariates were used to test if the interaction of time by gait condition at screening significantly predicted the neuropsychological test performances over time by controlling for the effect of age, gait condition at screening, time, the number of previous medical conditions, FAQ, volumes of the ventricles, and FDG-PET metabolism of the angular, temporal, and posterior cingulate regions. Disorders of abnormal gait were due to neurologic (51%; *N* = 20) or orthopaedic (49%; *N* = 19) conditions. We found a significant time by gait profile interaction on the MMSE, DSST, TMT-A, and TMT-B tests (see Fig. 1).

Regarding the DSST, we found a significant main effect of time (β = -0.03, SE = 0.01, *p* = 0.001) and a significant gait profile by time interaction (β = -0.19, SE = 0.04, *p* =  < 0.001). In particular, the slope for the abnormal gait group (β = -0.22, SE = 0.04, *p* =  < 0.001) was steeper than the one (β = -0.03, SE = 0.01, *p* = 0.009) of the normal gait group. That is to say that over time, aMCI patients with abnormal gait declined faster than the normal gait group on the DSST performance. The main effect of gait was not significant (β = -0.19, SE = 0.22, *p* = 0.408). The random effect ICC for the DSST was 0.85.

Regarding the TMT-A, we found a significant gait profile by time interaction (β = 0.17, SE = 0.04, *p* =  < 0.001). In particular, the slope in the abnormal gait group (β = 0.19, SE = 0.04, *p* < 0.001) was significant, whereas the slope for the normal gait group was not significant (β = 0.02, SE = 0.01, p = 0.083). That is to say that over time, only aMCI patients with abnormal gait declined on the TMT-A performance. The main effect of gait was not significant (β = 0, SE = 0.13, *p* = 0.994). The random effect ICC for the TMT-A was 0.67 (i.e., proportion of variance of the dependent variable explained by the random factors).

Concerning the TMT-B, we found a significant gait profile by time interaction (β = 0.23, SE = 0.04, *p* < 0.001). In particular, the slope for the abnormal gait group (β = 0.26, SE = 0.04, *p* < 0.001) was significant, whereas the slope for the normal gait group was not significant (β = 0.03, SE = 0.01, *p* = 0.066). That is to say that over time, only aMCI patients with abnormal gait declined on the TMT-B performance. The main effect of gait was not significant (β = 0.04, SE = 0.17, *p* = 0.827). The random effect ICC for the TMT-B was 0.75. Figure 1 shows the regression lines of these neuropsychological tests of interest.

Concerning the MMSE, we found a significant main effect of time (β = -0.06, SE = 0.02, *p* < 0.001) and a significant gait profile by time interaction (β = -0.15, SE = 0.05, *p* < 0.001). In particular, the slope for the abnormal gait group (β = -0.21, SE = 0.05, *p* < 0.001) was steeper than the one (β = -0.06, SE = 0.06, *p* < 0.001) of the normal gait group. That is to say that over time, aMCI patients with abnormal gait declined faster than the normal gait group on the MMSE performance. The main effect of gait was not significant (β = -0.06, SE = 0.16, *p* = 0.714). The random effect ICC for the TMT-B was 0.74. Figure 1 shows the regression lines of these neuropsychological tests of interest.

Conversely, the ADAS-13 and the RAVLT (immediate recall) declined over time independently of the gait profile variable. In particular, we found a significant main effect of time for the ADAS-13 (β = 0.09, SE = 0.02, *p* < 0.001) but the main effect of gait (β = 0.03, SE = 0.19, *p* = 0.866) and its interaction with time (β = -0.03, SE = 0.05, *p* = 0.491) were not significant. The random effect ICC for the ADAS-13 was 0.74. Lastly, the RAVLT (immediate recall) was affected by time (β = -0.05, SE = 0.02, *p* = 0.004) but not by gait profile (β = 0.24, SE = 0.2, *p* = 0.225) and its interaction with time (β = -0.03, SE = 0.05, *p* = 0.605). The random effect ICC for the RAVLT was 0.7.

For the complete list of the predictors/covariates analyses of all the neuropsychological tests included in this study see Supplementary Material 2.

### Effect of gait profiles on structural and functional imaging

RLMM regressions with covariates were used to test if the interaction of time by gait condition at screening significantly predicted neurophysiological changes over time by controlling for the effect of age, gait condition at screening, time, the number of previous medical conditions, FAQ, volumes of the ventricles, and FDG-PET metabolism of the angular, temporal, and posterior cingulate regions and dependent variable baseline scores. Four RLMM models were fitted for this purpose (Hippocampal model, in addition to the abovementioned covariates, adjusted also for baseline hippocampi volumetry; medial temporal lobe model, in addition to the abovementioned covariates, adjusted also for baseline medial temporal volumetry; ventricles model and FDG-PET model were adjusted only with the abovementioned covariates).

**Fig. 2:**
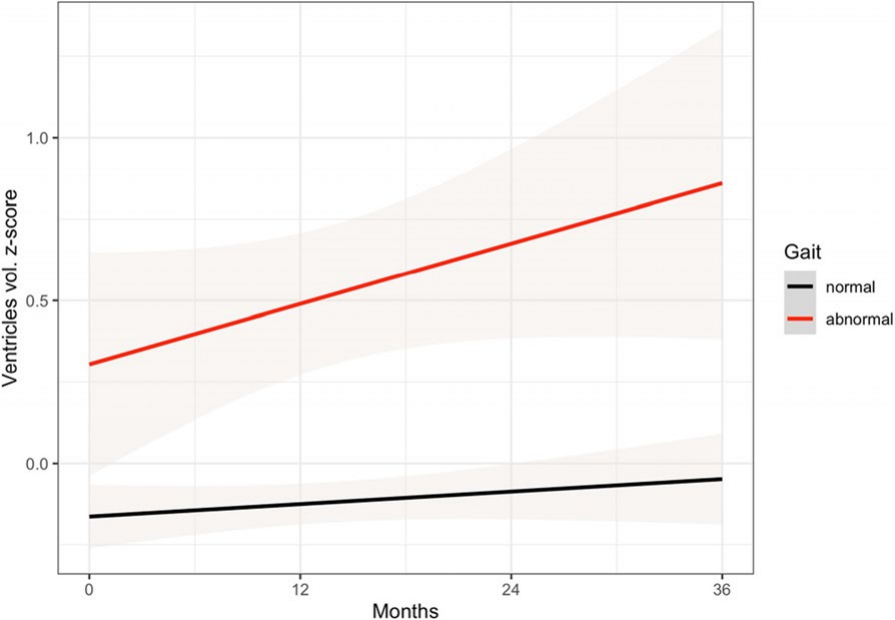
Trends in the amnestic Mild Cognitive Impairment group by gait profile on the ventricles size (volume). Positive z-score indicates larger ventricles. Gray shades in the plot represent the standard error of the regression line

For the ventricles model, we found a significant main effect of time (β = 0.1, SE = 0.01, *p* < 0.001) and a significant gait profile by time interaction (β = 0.02, SE = 0.01, *p* = 0.012). In particular, the slope for the abnormal gait group (β = 0.12, SE = 0.01, *p* < 0.001) was steeper than the one (β = 0.1, SE = 0.01, *p* < 0.001) of the normal gait group. That is to say that over time, aMCI patients with abnormal gait had a faster enlargement of the ventricles than the normal gait group. The main effect of gait was not significant (β = 0.01, SE = 0.02, *p* = 0.683). The random effect ICC for the TMT-B was 0.74. See Fig. 2 for this result.

For the hippocampal model, we only found a significant main effect of time (β = -0.13, SE = 0.01, *p* < 0.001). Regardless of the gait profile, hippocampi volumes declined over time. The main effect of gait profile was not significant (β = 0.08, SE = 0.04, *p* = 0.083). The ICC was 0.36.

For the medial temporal lobe model, we only found a significant main effect of time (β = -0.11, SE = 0.01, *p* < 0.001). Regardless of the gait profile, medial temporal regions volumes declined over time. The main effect of gait profile was not significant (β = 0.05, SE = 0.05, *p* = 0.34). The ICC was 0.32.

### Prognostic relevance of gait and gait-related measures on dementia conversion

A Cox proportional-hazards regression model was used to test the prognostic relevance of each of the five covariates of interest (gait profile, MMSE, DSST, TMT-A, and TMT-B) on conversion to dementia over the 36 months. Assumptions of the proportional hazards based on the scaled Schoenfeld residuals (all covariates and global model *p* > 0.05). This highlights a non-significant relationship between time and the residuals. In addition, the *p*-value (*p *< 0.001) for all three model tests (likelihood ratio, Wald, and score) were significant, indicating that the model is significant. Hence, we proceeded with the proposed analysis.

**Fig. 3:**
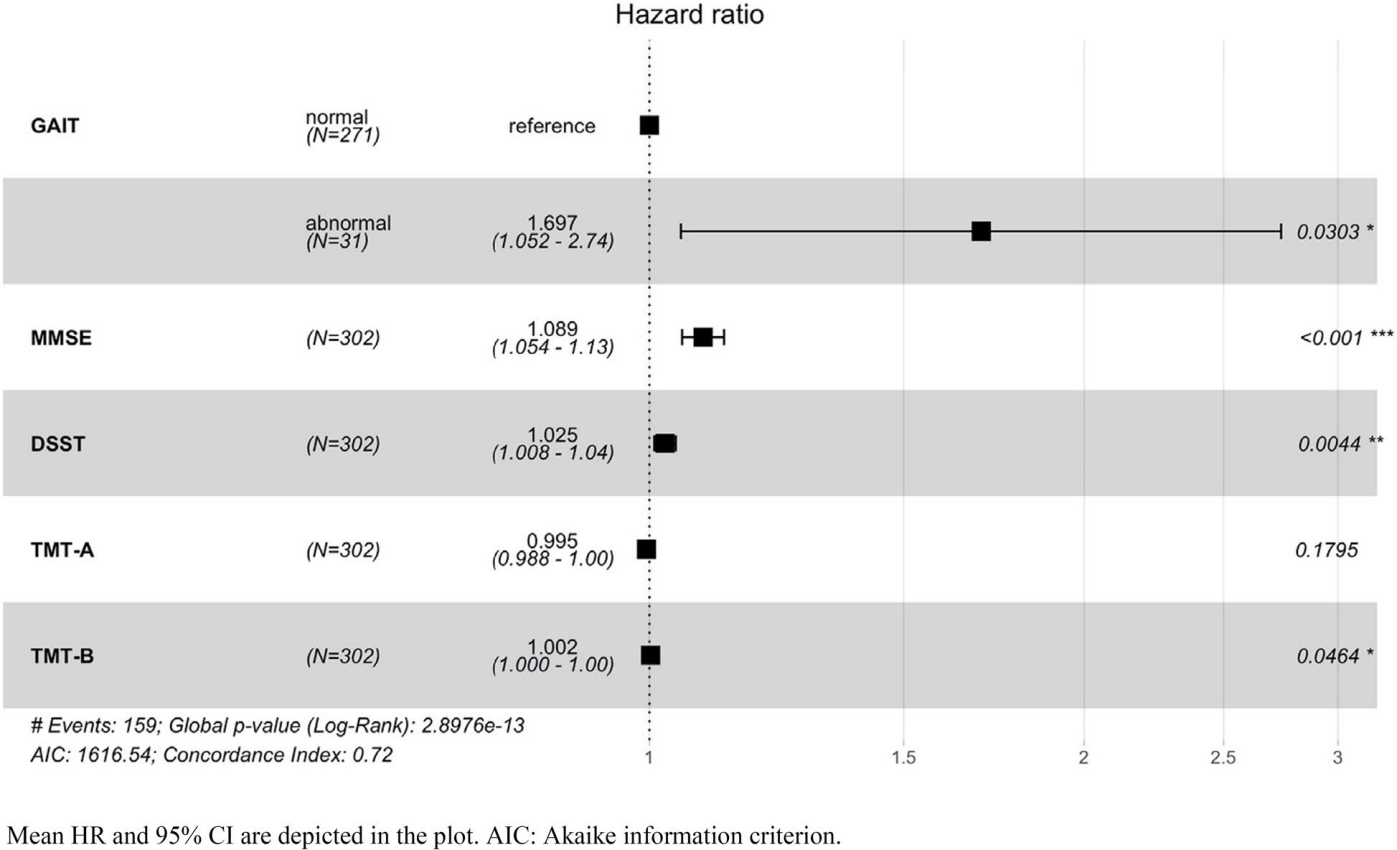
Forest plot of the Cox proportional-hazards model. A higher hazard ratio represents an increased risk of Alzheimer’s disease dementia conversion Mean HR and 95% CI are depicted in the plot. AIC: Akaike information criterion

We found that among the covariates included in the model the MMSE, DSST, and gait profile were significant. The presence of abnormal gait increases the risk of developing dementia in aMCI by 70% (HR = 1.7, *p* < 0.03), A decrease in one point of the MMSE increases the risk of developing dementia by 9% (HR = 1.09, *p* < 0.001), similarly a decrease in one point of the DSST increases the risk of developing dementia by 3% (HR = 1.03, *p* = 0.004). We also found a statistical tendency for the TMT-B (HR = 1, *p* = 0.046). The concordance index of 0.72 implies moderate concordance between risks and event time. Figure 3 shows the forest plot with HR, 95% CI of the HR, score test, and concordance index of this model.

## Discussion

The present study aimed at investigating the longitudinal trends in cognitive functions in the aMCI population, based on gait profiles at baseline and the prognostic relevance of gait and its related neuropsychological functions.

To our knowledge, there are no longitudinal studies in aMCI that evidence the influence of gait on a set of cognitive tests depending on gait disorders at a specific time point. This study analyzes the effect of the presence of gait disorders on repeated cognitive assessments and structural/functional brain imaging over time and evaluate the prognostic relevance of gait disorders and the significantly affected neuropsychological tests.

We found that gait abnormalities detected by a routinely neurological gait examination are associated with different trends in cognitive tests over time in aMCI. More precisely, when compared to the normal gait group, attention (DSST) and global cognition (MMSE) tests declined faster in the abnormal gait group compared to the normal gait group. Importantly, TMT part A and B uniquely declined over time in the abnormal gait group but not in the normal gait group. In addition, we showed that only ventricles volumes declined faster in the abnormal gait group, however this measure declined also for the normal gait group. Importantly, the presence of gait disorders (HR = 1.7) and the decline in the performance of two (MMSE, HR = 1.09; DSST, HR = 1.03) gait-related cognitive tests were associated with a greater risk of AD dementia conversion in the global aMCI ADNI population.

Our explorative analysis concerning the effect of the presence of gait disorders on a set of cognitive tests showed that some tests decline faster in aMCI with gait abnormalities than in aMCI with a normal gait, whereas other functions decline independently of this grouping variable. Crucially, we showed that psychomotor speed (TMT-A) and divided attention/cognitive flexibility (TMT-B) seem to be uniquely affected by gait abnormalities in aMCI. Less specific tests of cognitive functioning (MMSE, DSST), despite declining faster in the abnormal gait group, are not sensitive to gait disorders. Our findings regarding the link between gait and DSST and TMT-A are supported by previous studies on aging [6, 7, 12–16, 21] and MCI [27]. Concerning the results of TMT-B, our finding is in line with previous research on aging [6, 7] and MCI [27]. This suggests that psychomotor speed, attention, and executive functions are affected by gait and possibly by neuropathological changes in aMCI. A recent study found that additional frontal-executive dysfunction in aMCI increased the risk of dementia conversion compared with single-domain aMCI and that those patients showed diffuse cortical thinning, especially in the frontal areas [47]. Another research demonstrated that in aMCI the probability of developing dementia in the Alzheimer’s clinical syndrome a year later was significantly predicted by dysexecutive deficits [48]. Regarding global cognition, our result is in line with previous research that showed that gait abnormalities in healthy older people are longitudinally associated with global cognition performance [6, 7, 30], this has also been found in MCI [27]. Here, we extended the present literature by showing that such tests decline in aMCI with gait abnormalities and found that TMT-AB could be a sensible test to gait disorders in aMCI, rather than more general cognitive tests like MMSE or DSST. Indeed, the TMT-AB test is considered a core neuropsychological test to assess cognition and mobility in aging by the Canadian Consortium on Neurodegeneration in Aging [49].

Conversely, AD-related global cognition (ADAS-13) and auditory-verbal memory (immediate recall) decline over time independently of the presence of abnormal or normal gait. This suggests that in aMCI the decline in global cognition and memory is due to the presence of specific pathological changes potentially associated with AD [50], rather than with alterations in gait-related brain regions and functions.

Regarding brain alterations related to gait profiles, we showed that ventricles size increases faster in the abnormal compared to the normal gait group. FDG-PET, hippocampal size, and medial temporal lobe size declined regardless of the grouping variable. It could be argued that gait abnormalities are longitudinally associated with faster enlargement of the ventricles because of cortical brain atrophy [51]. For instance, a study [52] showed that enlargement of temporal horns and posterior portion of the ventricles is associated with gait instability in healthy older adults. It might be possible that the faster enlargement in the ventricles in the abnormal gait group is due to widespread cortical atrophy and possibly cognitive decline in executive functions/attention. Interestingly, the aMCI group with abnormal gait examination showed larger ventricles and lower FDG-PET metabolism at baseline, suggesting a link between gait disorder and ventricular size and temporoparietal brain metabolism.

In addition, we showed that the presence of gait disorders and MMSE and DSST score decline increased the risk of developing dementia. In accordance with our results, previous studies demonstrated that, in MCI, gait speed may be a sensitive marker of cognitive changes [28] and that individuals with deficits in gait velocity had a higher risk of progressing to dementia [29]. Furthermore, in aMCI, gait speed and gait variability may be markers for early detection of the likelihood of progression to AD [31]. Besides, slower baseline gait speed was associated with a higher hazard of developing aMCI/AD [32]. Recently, a large multicenter study [25] showed that higher gait variability could be a marker of AD. In addition to previous studies, we showed that gait-related measures decline, and in particular, the score of the MMSE and DSST tests, are risk markers of future dementia conversion. In contrast to our prediction, the TMT-A and TMT-B were not a prognostic marker for dementia conversion in aMCI despite being negatively affected by gait disorders. Indeed, the study by Kuate-Tegueu and co-authors [21] demonstrated that a low TMT-A score increased the risk of developing dementia; however, this study did not focus on aMCI but rather on healthy older persons.

Lastly, the presence of gait disorders hampers the autonomy (FAQ) of the individual. Indeed, we found that baseline reduced autonomy (higher FAQ score) and larger ventricles negatively influenced the DSST performance, whereas higher metabolism of the angular, temporal, and posterior cingulate regions (FDG-PET) positively influenced the score in the DSST performance. Conversely, the opposite directions were found for the TMT-A and TMT-B. The number of medical conditions at baseline was not associated with the decline in these tests, possibly because its effect is covered by the other covariates.

The findings of this work are also interesting considering the novel theoretical framework emphasizing the role of embodiment processes in aging. According to the embodiment theories, executive functions/attention and psychomotor speed are grounded in the ability to control and plan motor actions [53]. The notion that such functions are embodied in the sensorimotor system is also supported by a shared network of brain regions between motor and executive functions [3]. Indeed, some models of embodiment in aging suggest the importance of bodily information for the maintenance of cognitive abilities [54–56]. Spared motor processing in AD is thought to support cognitive abilities that are not affected by the disease in the early stages, such as motor planning and language comprehension [57]. Considering this theoretical proposal, we showed that gait in prodromal AD could affect neuropsychological functions related to motor execution and control of gait, raising the issue of the important role of bodily information on cognition. In addition, we found that the presence of gait disorders and executive functions/attention decline are risk factors for developing dementia. This hints that embodiment markers can be useful to detect individuals at greater risk of developing dementia even when the risk factor (i.e., gait and executive decline) is not a core clinical presentation of AD [19].

This study has certain limitations that must be considered. First, within the aMCI group, there is a strong numerical unbalance between normal gait and abnormal gait group sample size. Due to this disparity, appropriate statistical methods were used accordingly. Second, the neurological gait examination carried out according to the ADNI clinical protocol is categorical; a continuous outcome for walking performance could have improved our results and highlighted subtle changes also in other cognitive domains in aMCI [6]. Future research in the field of cognitive neuroscience could study embodiment with a specific motor task [57] in combination with neurophysiological instruments to deepen the understanding of embodiment markers in AD and aMCI. From the clinical point of view, future studies could design preventive cognitive training on executive functions/attention and psychomotor speed in aMCI with an abnormal gait. Indeed, gait and dual-task interventions should be tested to prevent motor and cognitive decline [58]. Finally, we propose that cognitive decline be monitored using DSST, TMT-A, and TMT-B in patients with aberrant gait aMCI so that test findings can detect probable neurophysiological alterations and signal faster cognitive decline and possible dementia.

## Conclusion

In conclusion, the study of the complex interaction between the motor and cognitive domains can help to better understand aging and neurodegenerative diseases and consequently to design innovative non-pharmacological interventions that target both domains. This study points out the impact of motor abilities and gait functioning on cognition supporting the interaction between physical and neuropsychological aspects. Importantly, clinicians and researchers should consider, in addition to memory, the importance of these functions for diagnostic, prognostic, and rehabilitative outcomes in aMCI.

## Supplementary Information

**Additional file 1.** 

**Additional file 2: Supplementary 2.** All cognitive tests and predictors.

## Data Availability

Data used in the preparation of this article were obtained from the Alzheimer’s Disease Neuroimaging Initiative (ADNI) database (adni.loni.usc.edu). As such, the investigators within the ADNI contributed to the design and implementation of ADNI and/or provided data but did not participate in the analysis or writing of this report. A complete listing of ADNI investigators can be found at: http://adni.loni.usc.edu/wp-content/uploads/how_to_apply/ADNI_Acknowledgement_List.pdf. The data that support the findings of this study are available from ADNI but restrictions apply to the availability of these data, which were used under license for the current study, and so are not publicly available. Data can be accessed through application to ADNI database (https://adni.loni.usc.edu/data-samples/access-data/).

## Abbreviations

ADNI: Alzheimer’s Disease Neuroimaging Initiative
AD: Alzheimer’s Disease
naMCI: Non-amnestic type
aMCI: Amnestic Mild Cognitive Impairment
BMI: Body Mass Index
GDS: Geriatric Depression Scale
FAQ: Functional Activities Questionnaires
ApoE4: Apolipoprotein E4
CVA: Cerebrovascular Accidents
FDG-PET: Average FDG-PET of angular, temporal, and posterior cingulate cortices
HP: Hippocampal Volume
MLT: Medial Temporal Lobe Volume
V: Ventricles Volume
WMH: White Matter Hyperintensities Volume
MRI: Magnetic Resonance Imaging
PET: Positron Emission Tomography
MMSE: Mini-Mental State Examination
ADAS-13: Alzheimer’s Disease Assessment Scale-Cognitive 13 items
RAVLT: Rey Auditory Verbal Learning Test
DSST: Digit Symbol Substitution Test
TMT-A: Trail Making Test part A
TMT-B: Trail Making Test part B
CDR: Clinical Dementia Rating
HR: Hazard Ratio
RLMM: Robust Linear Mixed-Effects Models
ICC: Intra-class Correlation Coefficient
