# Supplementary material for: Prognostic relevance of gait-related cognitive functions for dementia conversion in amnestic mild cognitive impairment
Source: BMC Geriatr. 2023 Jul 31;23:462. doi: 10.1186/s12877-023-04175-8 (PMC10388514; doi:10.1186/s12877-023-04175-8)
Supplement: Supplementary file 1 — Additional file 1. [file 12877_2023_4175_MOESM1_ESM.docx]

SUPPLEMENTARY 1

- Mini-Mental State Exam : The MMSE is a fully structured screening instrument in which orientation to place, orientation to time, registration, attention and concentration, recall, language and visual construction are evaluated. It is scored as the number of correctly completed items (from 0 to 30) with lower scores indicative of poorer performance and greater cognitive impairment.

- Alzheimer’s Disease Assessment Scale-Cognitive: The ADAS-COG (13 items) is a structured scale that evaluates memory, reasoning, language, orientation, ideational praxis, constructional praxis, spoken language, language comprehension, word finding difficulty, executive functions, and ability to remember test instructions. The test is scored in terms of errors (from 0 to 85), with higher scores reflecting poorer performance.

- Clock Drawing Test: In the “command” condition of this visuoperceptual constructional task, the subject is given a blank sheet of 8 1/2" X 11" paper and instructed to “Draw a clock, put in all of the numbers, and set the hands for 10 after 11.” After that task is completed, the “copy” condition ensues in which the subject attempts to copy a drawing of a clock with the hands set at ten past eleven. A quantitative score is derived for each drawing by adding the scores of three separate features: a maximum of 2 points is given for the integrity of the clock face; a maximum of 4 points for the presence and sequencing of the numbers; a maximum of 4 points for the presence and placement of the hands. A qualitative analysis can also be performed to note the presence of conceptual, perseverative, stimulus bound, and spatial arrangement errors.

- Digit Span Test : The Digit Span subtest from the Wechsler Adult Intelligence Scale-revised (WAIS-R) requires the subject to repeat sequences of single-digit numbers which are read aloud by the examiner. In the Forward condition, the subject must repeat the digits in the same order (the lengths of the sequences increase progressively from three to nine digits); in the Backward condition, the digits must be repeated in the reverse order (from two to eight digits). Two trials are presented for each sequence length. Testing is terminated when the subject misses both trials at a given sequence length. A point is awarded for each sequence correctly produced.

- Rey Auditory Verbal Learning Test: The RAVLT is a list learning task which assesses multiple cognitive parameters associated with learning and memory. On each of 5 learning trials, 15 unrelated nouns are presented orally at the rate of one word per second and immediate free recall of the words is elicited. The number of correctly recalled words on each trial is recorded. Following a 20-minute delay filled with unrelated testing, free recall of the original 15 word list is elicited. Finally, a yes/no recognition test is administered which consists of the original 15 words and 15 randomly interspersed distracter words. The number of target “hits” and false positive responses are recorded.

- Trail Making Test: Parts A and B: Part A consists of 25 circles numbered 1 through 25 distributed over a white sheet of 8 1/2" X 11" paper. The subject is instructed to connect the circles with a drawn line as quickly as possible in ascending numerical order. Part B also consists of 25 circles, but they are either numbered (1 through 13) or contain letters (A through L). Now the subject must connect the circles while alternating between numbers and letters in an ascending order. The subject's performance is judged in terms of the time required to complete each trail and by the number of errors of commission and omission.

- Digit Symbol Substitution Test: This subtest from the WAIS-R consists of 93 small blank squares (presented in seven rows) each randomly paired with one of nine numbers (1 to 9) printed directly above it. Above the row of blank squares is a printed “key” that pairs each of the numbers 1 through 9 with an unfamiliar symbol. Following a short series of practice trials, the subject must use the key to fill in the blank squares in order (working left to right across the rows) with the symbol that is paired with the number above it, working as quickly as possible for 90 seconds. The number of blank squares filled in correctly within the time limit is the measure of interest (Maximum raw score = 93).
