# Supplementary material for: Prognostic relevance of gait-related cognitive functions for dementia conversion in amnestic mild cognitive impairment
Source: BMC Geriatr. 2023 Jul 31;23:462. doi: 10.1186/s12877-023-04175-8 (PMC10388514; doi:10.1186/s12877-023-04175-8)
Supplement: Supplementary file 2 — Additional file 2: Supplementary 2. All cognitive tests and predictors. [file 12877_2023_4175_MOESM2_ESM.docx]

SUPPLEMENTARY 2

All cognitive tests and predictors

| Estimate | Std. Error | t value | test | p_value | predictors |
| --- | --- | --- | --- | --- | --- |
| -0.12 | 0.05 | -2.27 | ADAS13 | 0.024 | Intercept |
| 0.03 | 0.19 | 0.17 |  | 0.866 | Gait |
| 0.09 | 0.02 | 5.85 |  | < 0.001 | Time |
| 0.02 | 0.05 | 0.33 |  | 0.739 | Age bl |
| 0.28 | 0.03 | 10.62 |  | < 0.001 | FAQ bl |
| 0.07 | 0.05 | 1.29 |  | 0.2 | Medical bl |
| 0.05 | 0.05 | 0.89 |  | 0.377 | Ventricles bl |
| -0.25 | 0.05 | -4.8 |  | < 0.001 | FDG bl |
| -0.03 | 0.05 | -0.69 |  | 0.491 | Gait by time |
| 0.12 | 0.04 | 2.77 | MMSE | 0.006 | Intercept |
| -0.06 | 0.16 | -0.37 |  | 0.714 | Gait |
| -0.06 | 0.02 | -3.61 |  | < 0.001 | Time |
| -0.04 | 0.05 | -0.86 |  | 0.393 | Age bl |
| -0.38 | 0.03 | -12.92 |  | < 0.001 | FAQ bl |
| 0.08 | 0.05 | 1.63 |  | 0.104 | Medical bl |
| 0.02 | 0.05 | 0.52 |  | 0.604 | Ventricles bl |
| 0.19 | 0.05 | 4.15 |  | < 0.001 | FDG bl |
| -0.15 | 0.05 | -2.72 |  | 0.007 | Gait by time |
| -0.02 | 0.05 | -0.45 | RAVLT_immediate | 0.652 | Intercept |
| 0.24 | 0.2 | 1.22 |  | 0.225 | Gait |
| -0.05 | 0.02 | -2.9 |  | 0.004 | Time |
| -0.03 | 0.06 | -0.53 |  | 0.595 | Age bl |
| -0.23 | 0.03 | -7.57 |  | < 0.001 | FAQ bl |
| -0.08 | 0.06 | -1.49 |  | 0.139 | Medical bl |
| -0.1 | 0.06 | -1.81 |  | 0.072 | Ventricles bl |
| 0.18 | 0.06 | 3.27 |  | 0.001 | FDG bl |
| -0.03 | 0.05 | -0.52 |  | 0.605 | Gait by time |
| 0.04 | 0.06 | 0.7 | RAVLT_learning | 0.483 | Intercept |
| 0.02 | 0.21 | 0.07 |  | 0.941 | Gait |
| -0.02 | 0.03 | -0.63 |  | 0.531 | Time |
| 0.06 | 0.06 | 1.06 |  | 0.29 | Age bl |
| -0.19 | 0.04 | -4.43 |  | < 0.001 | FAQ bl |
| -0.12 | 0.06 | -1.99 |  | 0.048 | Medical bl |
| 0 | 0.06 | 0.01 |  | 0.994 | Ventricles bl |
| 0.11 | 0.06 | 1.92 |  | 0.057 | FDG bl |
| 0.1 | 0.08 | 1.2 |  | 0.231 | Gait by time |
| 0.01 | 0.05 | 0.12 | RAVLT_forgetting | 0.901 | Intercept |
| 0.21 | 0.18 | 1.16 |  | 0.248 | Gait |
| -0.05 | 0.03 | -1.85 |  | 0.065 | Time |
| 0.01 | 0.05 | 0.13 |  | 0.895 | Age bl |
| 0.01 | 0.04 | 0.34 |  | 0.736 | FAQ bl |
| 0.03 | 0.05 | 0.5 |  | 0.62 | Medical bl |
| -0.07 | 0.05 | -1.39 |  | 0.166 | Ventricles bl |
| -0.06 | 0.05 | -1.17 |  | 0.245 | FDG bl |
| 0.05 | 0.08 | 0.66 |  | 0.51 | Gait by time |
| 0.05 | 0.06 | 0.83 | DSST | 0.408 | Intercept |
| -0.19 | 0.22 | -0.87 |  | 0.384 | Gait |
| -0.03 | 0.01 | -2.59 |  | 0.01 | Time |
| 0.01 | 0.06 | 0.16 |  | 0.872 | Age bl |
| -0.12 | 0.02 | -5.32 |  | < 0.001 | FAQ bl |
| -0.03 | 0.06 | -0.56 |  | 0.579 | Medical bl |
| -0.15 | 0.06 | -2.46 |  | 0.015 | Ventricles bl |
| 0.31 | 0.06 | 5.13 |  | < 0.001 | FDG bl |
| -0.19 | 0.04 | -4.84 |  | < 0.001 | Gait by time |
| 0.11 | 0.05 | 2.42 | CDT | 0.016 | Intercept |
| 0.15 | 0.17 | 0.91 |  | 0.366 | Gait |
| -0.03 | 0.02 | -1.41 |  | 0.16 | Time |
| -0.02 | 0.05 | -0.37 |  | 0.713 | Age bl |
| -0.17 | 0.04 | -4.34 |  | < 0.001 | FAQ bl |
| -0.02 | 0.05 | -0.32 |  | 0.748 | Medical bl |
| -0.02 | 0.05 | -0.43 |  | 0.671 | Ventricles bl |
| 0.21 | 0.05 | 4.25 |  | < 0.001 | FDG bl |
| -0.08 | 0.08 | -1.02 |  | 0.309 | Gait by time |
| -0.04 | 0.06 | -0.62 | DSF | 0.534 | Intercept |
| 0.4 | 0.23 | 1.74 |  | 0.084 | Gait |
| -0.03 | 0.02 | -1.15 |  | 0.251 | Time |
| -0.08 | 0.07 | -1.21 |  | 0.229 | Age bl |
| -0.09 | 0.04 | -2.27 |  | 0.023 | FAQ bl |
| -0.05 | 0.07 | -0.72 |  | 0.473 | Medical bl |
| 0.04 | 0.06 | 0.6 |  | 0.55 | Ventricles bl |
| 0.12 | 0.06 | 1.84 |  | 0.068 | FDG bl |
| -0.02 | 0.08 | -0.3 |  | 0.766 | Gait by time |
| -0.06 | 0.05 | -1.14 | DSB | 0.254 | Intercept |
| 0.24 | 0.2 | 1.22 |  | 0.223 | Gait |
| -0.03 | 0.02 | -1.08 |  | 0.282 | Time |
| -0.06 | 0.06 | -1.05 |  | 0.294 | Age bl |
| -0.09 | 0.04 | -2.31 |  | 0.021 | FAQ bl |
| 0.05 | 0.06 | 0.95 |  | 0.344 | Medical bl |
| 0.01 | 0.06 | 0.23 |  | 0.819 | Ventricles bl |
| 0.26 | 0.06 | 4.77 |  | < 0.001 | FDG bl |
| -0.1 | 0.07 | -1.37 |  | 0.172 | Gait by time |
| -0.2 | 0.04 | -5.6 | TMT-A | < 0.001 | Intercept |
| 0 | 0.13 | 0.01 |  | 0.994 | Gait |
| 0.02 | 0.01 | 1.73 |  | 0.084 | Time |
| 0.01 | 0.04 | 0.24 |  | 0.81 | Age bl |
| 0.08 | 0.02 | 3.57 |  | < 0.001 | FAQ bl |
| 0.04 | 0.04 | 1.03 |  | 0.304 | Medical bl |
| 0.1 | 0.04 | 2.65 |  | 0.009 | Ventricles bl |
| -0.17 | 0.04 | -4.56 |  | < 0.001 | FDG bl |
| 0.17 | 0.04 | 4.42 |  | < 0.001 | Gait by time |
| -0.13 | 0.05 | -2.8 | TMT-B | 0.006 | Intercept |
| 0.04 | 0.17 | 0.22 |  | 0.827 | Gait |
| 0.03 | 0.01 | 1.84 |  | 0.067 | Time |
| -0.02 | 0.05 | -0.42 |  | 0.677 | Age bl |
| 0.09 | 0.03 | 3.38 |  | 0.001 | FAQ bl |
| 0.01 | 0.05 | 0.28 |  | 0.78 | Medical bl |
| 0.15 | 0.05 | 3.14 |  | 0.002 | Ventricles bl |
| -0.31 | 0.05 | -6.67 |  | < 0.001 | FDG bl |
| 0.23 | 0.04 | 5.56 |  | < 0.001 | Gait by time |
